# Association of Helicobacter Pylori Infection and Ectopic Pregnancy 

**Published:** 2016-06

**Authors:** Fariba Nanbakhsh, Tahereh Behrouzi-Lak, Mahsa Tabean, Sima Oshnouei, Pooya Mazlumi

**Affiliations:** Reproductive Health Research Center, Department of Obstetrics and Gynecology, Urmia University of Medical Sciences, Urmia, Iran

**Keywords:** Ectopic Pregnancy, IgG, CagA Antibody, Helicobacter Pilory

## Abstract

**Objective:** To evaluate the importance of cytokine type in embryo implantation in uterus specified and activated macrophages interfere the tube movements and embryo retention in uterine tubes by smooth muscle relaxation and disrupting ciliary function. Therefore, increased risk of infection with HP during pregnancy, we investigated relation between Helicobacter pylori (HP) infection and prevalence of ectopic pregnancy (EP) in this study.

**Materials and methods:** This is cross-sectional study from March 2012 to May 2013. Totally 207 women were enrolled randomly from which 101 had EP (Case group) and 106 were selected as control group with normal pregnancy. A 2-cc blood sample was taken from each patient to evaluate the specific IgG titer by ELISA method. All results of samples with positive H. pylori IgG were assayed for anti-CagA, IgG antibodies. A questionnaire was filled for each subject. The associations between CagA positive cases with odds of Ectopic pregnancy incidence were analyzed by using SPSS software, ver. 19 (Chicago, IL, USA).

**Results:** Mean (± SD) of age were 21.0 ± 5.78 and 30.78 ± 5.10 years for cases and controls group respectively. These groups didn’t show any significance difference in age and parity.H. pylori IgG antibodies were positive among 99 and 103 (98.2% vs. 97.2%) in women with EP and normal pregnancy respectively. Relationship between IgG status and EP was not significant (OR = 1.31: 95% CI = 0.7-2.52, Pvalue = 0.37). In particular anti-CagA antibodies were positive among 45 and 39(45.92% vs. 36.97%) in women with EP and normal pregnancy respectively. Among women with CagA positive strains had higher odds of Ep (OR = 1.46: 95% CI = 0.8-2.65, Pvalue = 0.18), but it wasn’t significant.

**Conclusion:** According to the result of this study there was not any association between HP infection and Ectopic pregnancy. We recommend more studies with larger sample size for determining the effect of CagA positive strains on EP.

## Introduction

Ectopic pregnancy (EP) is the implantation of blastocyst in a place other than uterine endometrial lining and its most commonly reported from uterine tubes. Regardless of early diagnosed and treated, EP associated-hemorrhage is still one of the main reasons of maternal death in first trimester and comprises 6% of pregnancy -related deaths. Its prevalence is estimated 2% of pregnancies (   1 ). Since the mid-twentieth century, increased pelvic infection outbreaks were accompanied by increased outbreak of ectopic pregnancy (2). 

Abnormalities in uterine tube movements leading to dysfunctions, and damage to uterine tube cilia which delays or blocks oocyte passage has been suggested to be the main reason of EP.

Pelvic infections like uterine tube inflammation whit chlamydia, gonorrhea and especially recurrent infections changing uterine tube function which may lead to obstruction and pelvic adhesions (3). Probably, chlamydia infection increase the implantation in uterine tubes via production of PROKR2 protein (4). Also, history of previous EP,uterine tube surgery, infertility and cervix infections associate with high levels of EP risk, and various sexual partners, smoking and previous abdominal and pelvic surgeries associate with moderate levels of EP risk (5). 

*Helicobacter pylori *(HP) is a gram negative, catalase, and urease and oxidase positive bacteria. Infection with HP is the most common chronic infection reported in human. Oral-oral route, oral-fecal route and medical interventions such as endoscopy are the most common ways of person to person transmission. Also, increased fast food consumption and poor social and economical conditions facilitates the transmission (6). Prevalence is reported 80- 50 %in developing and developed countries respectively. Helicobacter pylori infection damages stomach by releasing enzymes, toxins and attaching gastric mucosa and causing morphological and functional changes in epithelial cells (7). In animal models, Helicobacter typhlonius was found in mice genital organ as a new member of this family using PCR method (8).Vaginal environment is similar to gastric environment in terms of pH and ecologic properties. The bacteria uses it microairophilic ability, can colonize and infect the person without any special clinical feature (9). There is a close relation between serum and cervival mucosa levels of HP antibody titers. Presence of this antibody plays an important role in infertility, inhibiting sperm penetration. Several humoral and cellular immunity variations in pregnancy may lead to *Helicobacter pylori* infection (10). 

Cytokines are immune-modulator molecules, which show immune response and can effect reproductive procedures, such as ovulation, implantation and delivery (11). 

In early pregnancy, endometrial tissue grow is associated with TH1 response which produces pre-inflammatory cytokines. During pregnancy TH1 response changes to TH2 and again in late pregnancy TH1 response causes uterine contraction and delivery. Therefore Th2 secreted cytokines like Interlukin-10 supports pregnancy progress, while TH1 secreted cytokines like TNF-alpha and IL1-beta have roles in implantation (12). Pre-inflammatory cytokines including TNF-alpha, IFN-gamma, Interlukin 1,6 and 8, Leukemia inhibiting factor (LIF) induce apoptosis, thus removing intercellular micro organisms (13). With its regulatory and stimulatory effect on embryo implantation, LIF triggers trophoblasts to invade endometer. The latter action is necessary in blastocyst implantation in uterine. LIF chemically calls macrophages and limits immigration of killer cells. Infection caused inflammation in uterine tubes, increases the LIF level. So, presence of macrophage and lack of natural killers cells are the main triggers of EP (14). TH1 response is dominant in HP infections. During the infection, producing antigenic products like acute phase proteins, urease and lipopolysaccarides increases production of inflammatory cytokines like interlukin-1, TNF-alpha and interlukin-8 (15). In America and Europe HP causes a TH1 response that is not capable of microorganism elimination, damages gastric mucosa and increases systemic inflammation. High levels of pre-inflammatory cytokines damages organs other than stomach. But in developing countries the response changes to TH2, due to intestinal worm and other parasitic infections, which produces non-inflammatory cytokines (16).

The main genetic difference between species of HP is presence of CagA gene. CagA is not cytotoxic but antigenic, and its antibody can be detected in gastric tissue and serum. CagA is present in 60-70 percent of species, and these species are strong producers of interlukin-8. Thus infection with these species are associated with higher levels of inflammatory mediators (17). Elimination of HP using a 4 drug regimen in infected female mice increased fertility, fetus number, fetus weight and total pregnancy status (18). 

Chronic salpingitis is present in almost 90% of EP cases (19). In chronic salpingitis, producing growth factors, cytokines, TNF-alpha and interlukin-1, macrophages can help implantation in uterine tubes. Furthermore, activated macrophages causes an increase in progesterone which interferes the tube movements and embryo retention in uterine tubes by smooth muscle relaxation and disrupting ciliary function (20). 

**Table 1:** Age and parity distribution in 2 study groups

Variables		Cases	Controls	p value
**Age (mean ± SE)**		**28.31 ± 2.81**	**27.14 ± 1.94**	**0.73**
**Parity**		**n (%)**	**n (%)**	**0.13**
	**1**	**16 (15.8)**	**29 (27.4)**	
	**2**	**49 (48.5)**	**45 (42.9)**	
	**3**	**21 (20.8)**	**21 (19.8)**	
	**4**	**8 (7.9)**	**5 (4.7)**	
	**5**	**7(6.9)**	**6 (5.7)**	

Considering the importance of cytokine type in embryo implantation in uterine, increased risk of infection with HP during pregnancy and change in level and type of antibody, in the current study we investigated relation between HP infection and prevalence of EP in a cross-sectional study in a teaching hospital.

## Materials and methods

Two hundred and seven women enrolled to this cross-sectional study from March 2012 to May 2013 in obstetrics and gynecology clinic of ShahidMotahhari teaching hospital of Urmia University of Medical Sciences; in which 101woman had EP and the rest 106 women is selected as control with normal pregnancy. The study was confirmed by the Ethics Committee of Urmia University of Medical Sciences (ir.umsu.rec.1392.68). 

Patients with history of pelvic infections, previous EP, smoking, various sex partners and other EP causes were excluded from this group.

A complete history taking was followed by physical examination and abdominopelvic ultrasonography. Patient’s data, including, age, number of gestation, delivery and gynecologic disorders history were collected using data forms. Then to assess the level of antibodies in serum, 2 ml blood sample was taken from all patients. The samples were first tested for Helicobacter pylori IgG using ELISA method and if the initial results were positive, secondary test for CagA IgG was performed. Serologic studies were done by using antibody kits (H pylory G plus, Germany and H. pylori CagA-Ag, Germany). To evaluate the relation between outcome and risk factors Fisher exact test were used. If there were a relation between HP infection and EP increased risk and prevalence, logistic regression model was used.

## Results

Totally 207 pregnant women in two groups, one with 101 EP patients and the other with 207 term pregnant patients were selected for the study. Table 1 shows age and parity distribution in 2 groups. These groups didn’t show any significance difference in age and parity.

IgG antibody against HP level was determined in 99 patients (98.2%) in case group and 103 patients (97.2%) in control group. Seventy two patients with EP (72.7%) and 69 in control group (67%) were reported positive for HP IgG. Ninety eight patients with EP (97.03%) and all normal pregnant patients were assessed for HP CagA antibody. From all, 45 EP patients (45.92%) and 39 normal pregnant patients (36.97%) were reported as positive. (Table 2).

Positive IgG and CagA antibody levels against HP were not significantly associated with increased risk of EP. (OR = 1.31: 95% CI = 0.7-2.52, p value = 0.37, OR = 1.46: 95% CI = 0.8-2.65, p value = 0.18).

**Table 2:** Distribution of IgG and CagA markers level against Helicobacter pylori in ectopic pregnant patients

**Variables**		Cases **n (%)**	Controls **n (%)**	**OR (95% CI)**	**p value**
**IgG status**	**Positive**	**72 (72.73)**	**69 (66.99)**	**1.31 (0.7-2.52)**	**0.37**
	**Negative**	**27 (27.27)**	**34 (33.01)**		
**CagA status**	**Positive**	**45 (45.92)**	**39 (36.79)**	**1.46 (0.8-2.65)**	**0.18**
	**Negative**	**53 (54.08)**	**67 (63.21)**		

## Discussion

Positive IgG and CagA antibody levels against HP were not significantly associated with increased risk of EP.

Althoughthe number of patients with positive test in case group was more than control group, but there was no significant relation. More studies with bigger sample size may change the results.

In recent years articles hypothesizing the probability of a relation between fertility problems and HP infection, were published. Rossi discusses the negative effect of HP infection on implantation and fetal weight, and states increase in macrophage activity and CD4^+^, CD8^+^ and INF-gamma as causes of bacteria stimuli. Infection with HP triggers activity of uterine immune cells and omits this hypothesis that HP can activate Th1, Th1 and Th2 imbalance, so causing above mentioned side effects (21). 

Chichlowski et al in a study on HP infection in mice and its effect on reproductive system, reported transient bacteriemia in tissues and then colonization in digestive organs. They also mentioned the probability of transmission via placenta, weight loss and abortion. Fertility reduced in HP infection and treatment with a 4 drug regimen causes and increase in fertility and fetus number (9). Th1 response in endometrial level is the suggested mechanism for these symptoms, but there is no enough evidence to prove it.

Khalili emphasized that on comparing infection in fertile and infertile women, that HP infection ratio in infertile women is 67% which doesn’t have any significant relation with infertility (P = 0.6). He also reported that HP antibody prevalence in tube-factor infertile people comparing to other infertility causes like PCO, was significantly higher (P = 0.05) (22). 

On the other hand, Figura et al. in a similar study compared 167 infertile women with 837 control, reported the antibody titer level significantly higher (P < 0.05). They also pointed that follicular fund sample containing HP antibody is responsible of disorder in fetal cells. HP antibody was positive in follicular fluid of all infected infertile patients, 50% of sperm samples and some of vaginal discharges (16). 

In a study performed by Granot. I et al. immune response in addition to active Th1 inflammatory response in coordination with embryo implantation in uterus and its effect on EP was mentioned. It was approved in this study that, local damage via inflammatory response and accelerating residual cell growth increases the acceptance of uterine for implantation. In a group that had implantation failure more than 7 times, to induce local inflammation, uterine biopsies were performed in proliferative phase and IVF results was increased by means of implantation from 4% to 11% (20). 

Infection with HP affecting sperm quality, increasing inflammatory reaction and immunological reaction between bacteria and sperm antigen in CagA positive men, decreasing sperm movement and increasing apoptosis and necrosis were effective in fertility (23). 

In all the latest articles, the relation between HP infections, intrauterine embryo growth limitation, early abortion and reduced sperm quality were shown by increased inflammatory factors and changes in their balance. Granot. I et al. reported increase in implantation due to increase in local inflammation (20).

In this study, PositiveIgG and CagA antibody levels against HP were not significantly associated with increased risk of EP.

Although the number of patients with positive test in case group was more than control group, but there was no significant relation. More studies with bigger sample size may change the results.
